# Sequential versus concurrent neoadjuvant immunochemotherapy in locally advanced esophageal squamous cell carcinoma: a randomized, controlled, open-label, phase 2 trial (HCHTOG1906)

**DOI:** 10.3389/fimmu.2026.1770662

**Published:** 2026-05-07

**Authors:** Yan Zheng, Jiwei Wu, Lingdi Zhao, Yaxing Shen, Guanghui Liang, Keting Li, Quanli Gao, Wenqun Xing

**Affiliations:** 1 State Key Laboratory of Metabolic Dysregulation & Prevention and Treatment of Esophageal Cancer, The Affiliated Cancer Hospital of Zhengzhou University&Henan Cancer Hospital, Zhengzhou, Henan, China; 2 Department of Thoracic Surgery, The Affiliated Cancer Hospital of Zhengzhou University&Henan Cancer Hospital, Zhengzhou, Henan, China; 3 Department of Immunotherapy, Cancer Hospital Affiliated to Zhengzhou University and Henan Cancer Hospital, , Zhengzhou, Henan, China; 4 Department of Thoracic Surgery, Zhongshan Hospital Affiliated to Fudan University, Shanghai, China; 5 Department of Thoracic Surgery, The Affiliated Chest Hospital of Zhengzhou University/Henan Chest Hospital, Zhengzhou, Henan, China

**Keywords:** esophageal squamous cell carcinoma, neoadjuvant immunochemotherapy, pathological complete response, PD-1 inhibitor, timing sequence

## Abstract

**Background:**

Neoadjuvant chemotherapy combined with PD-1 inhibitors has shown application potential in esophageal squamous cell carcinoma (ESCC), however, optimization strategies for administration timing (sequential vs. concurrent) still lack support from prospective evidence.

**Methods:**

This open-label, phase II randomized controlled trial enrolled a total of 70 patients with resectable locally advanced ESCC from July 2019 to June 2023. Patients were randomized 1:1 to the sequential group (paclitaxel/cisplatin on day 1, toripalimab on day 3) or the concurrent group (all drugs on day 1). The primary outcome was pathological complete remission (pCR). The secondary outcomes were to explore the safety of chemotherapy combined with toripalimab as neoadjuvant treatment under different administration sequences, evaluating treatment-related adverse events (AEs), overall survival (OS) and disease-free survival (DFS).

**Results:**

All 70 patients completed neoadjuvant treatment, and 54 patients underwent surgery. The overall pCR rate was 22.2% (12/54), with no significant difference between the sequential group and the concurrent group (17.8% vs. 26.9%, P = 0.636). In terms of safety, the incidence of grade 3–5 TRAEs was 14.2% (10/70). The concurrent group had significantly higher incidences of nausea and diarrhea (P<0.05). A total of 6 treatment-related deaths occurred, with 5 cases in the concurrent group and 1 case in the sequential group. There were no statistically significant differences in OS (P = 0.780) or DFS (P = 0.632) between the two groups.

**Conclusions:**

There was no significant difference in pCR or survival outcomes between sequential and concurrent immunochemotherapy, but the concurrent strategy was associated with increased risks of nausea, diarrhea, and fatal immune-related adverse events (irAEs).

## Introduction

Esophageal Cancer (EC) ranks 9th in incidence among all malignant tumors and 6th in cancer-related mortality (1, 2). In recent years, the clinical application of immune checkpoint inhibitors (ICIs), particularly anti-programmed death 1 (PD-1) antibodies, has revolutionized the treatment landscape of EC. These agents have successively obtained approvaled for second-line and first-line indications in esophageal squamous cell carcinoma (ESCC), and several investigator-initiated trials (IITs) have also revealed their potential survival benefits in neoadjuvant setting (3–8). However, there remains many uncertainties and controversies regarding the combination mode of PD-1 monoclonal antibodies with chemotherapy (9). Preclinical studies have confirmed that chemotherapy can exert a synergistic anti-tumor effect with ICIs through mechanisms such as inducing immunogenic cell death (ICD) and depleting myeloid-derived suppressor cells (MDSCs) (10–13). Derived from the dynamic change characteristics of the tumor immune microenvironment (TIME) induced by chemotherapy, such as the dynamic variation in PD-1 expression levels on the surface of CD8+ T cells (14–17). This feature suggested that ICI intervention might have a time-dependent “immune sensitive window”. Nevertheless, in the context of neoadjuvant treatment, the core issue of the administration timing of chemotherapy and ICIs remains unresolved.

In recent years, studies related to administration timing have achieved preliminary progress in animal experiments and clinical trials, with most results supporting that “sequential chemoimmunotherapy” can improve efficacy (18–21). In lung squamous cell carcinoma, a study by He et al (22). demonstrated that the synergistic anti-tumor effect of low-dose sequential chemotherapy combined with anti-PD-1 antibodies was significantly superior to that of the concurrent administration regimen; in the field of non-small cell lung cancer, a study conducted by Huo et al (23). further showed that the objective response rate (ORR: 68% vs. 37%) and progression-free survival (PFS) of administering anti-PD-1 antibodies 3 days after chemotherapy were significantly better than those of the concurrent regimen. These studies indicate that the treatment timing of chemoimmunotherapy may have a critical impact on efficacy and safety. In contrast, in the field of ESCC, most current clinical trials of neoadjuvant chemoimmunotherapy still adopt the concurrent administration mode (24, 25),only our previous randomized phase II study suggested (26) that administering anti-PD-1 antibodies 2 days after chemotherapy might improve the pCR rate (every-other-day group vs. same-day group: 36% vs. 7%), but due to the limitation of sample size (15 patients in each group), the preset statistical significance was not achieved (p=0.079). Therefore, the sample size was enlarged to clarify the exact value and safety of the sequential strategy in neoadjuvant treatment of ESCC.

To clarify the uncertainties observed in our prospective, randomized phase II clinical trial, we increased sample size The study aimed to optimize the combination mode of neoadjuvant chemoimmunotherapy through a head-to-head comparison of sequential and concurrent strategies, providing evidence to guide future clinical practice and larger-scale phase III trials.

## Methods

### Inclusion criteria

- Aged ≥18 years and ≤70 years, regardless of gender;
- Pathologically confirmed thoracic esophageal squamous cell carcinoma (ESCC);
- No distant metastasis confirmed by imaging examinations, and esophageal cancer deemed resectable or potentially resectable by thoracic surgery specialists after consultation;
- Eastern Cooperative Oncology Group (ECOG) performance status score of 0–1;
- Clinical Staged as II, III, or IVa according to the 8th edition of the American Joint Committee on Cancer (AJCC) Cancer Staging Manual;
- Sufficient organ function, defined as: (1), No need for growth factor or blood component support within 2 weeks before enrollment; (2), Cardiac function: No heart disease or coronary heart disease, with a cardiac function grade of 1–2; (3), Hepatic function: Total bilirubin (TBIL) ≤2 times the upper limit of normal (ULN), aspartate aminotransferase (AST) ≤2.5 ULN, alanine aminotransferase (ALT) ≤2.5 ULN; (4), Renal function: Creatinine (Cr) ≤1.25 ULN;
- Normal blood pressure, or well-controlled blood pressure within the normal range using antihypertensive drugs for patients with hypertension;
- Fasting blood glucose ≤8 mmol/L controlled by hypoglycemic drugs for patients with diabetes;
- No other severe diseases that conflict with this protocol (e.g., autoimmune diseases, immunodeficiency, organ transplantation);
- No history of other malignant tumors;
- Women of childbearing age must have a negative serum pregnancy test within 7 days before enrollment, and all subjects of childbearing age must use appropriate contraceptive measures during the trial and within 6 months after the trial.

### Exclusion criteria

- Pregnant or lactating women;
- History of severe infectious diseases within 4 weeks before enrollment;
- Patients with bronchial asthma requiring intermittent use of bronchodilators or medical intervention;
- Use of immunosuppressants due to comorbid diseases before enrollment, with a dosage equivalent to ≥10 mg/day of oral prednisone for more than 2 consecutive weeks;
- Clinically significant cardiovascular and cerebrovascular diseases, including but not limited to severe acute myocardial infarction, unstable or severe angina pectoris, coronary artery bypass grafting, congestive heart failure, ventricular arrhythmias requiring medical intervention, or left ventricular ejection fraction (LVEF) <50% within 6 months before enrollment;
- Severe allergic diathesis;
- Severe mental disorders;
- Abnormal coagulation function [prothrombin time (PT) >16 s, activated partial thromboplastin time (APTT) >53 s, thrombin time (TT) >21 s, fibrinogen (Fib) <1.5 g/L], bleeding tendency, or ongoing thrombolytic or anticoagulant therapy;
- Past or current pulmonary fibrosis, interstitial pneumonia, pneumoconiosis, radiation pneumonitis, severe impairment of pulmonary function, or other similar pulmonary conditions;

### Random assignment

Patients were randomly assigned at a 1:1 ratio and allocated to the sequential group or concurrent group according to the enrollment sequence. The random assignment protocol was generated by computer, using a simple randomization method. The assignments were placed in sealed envelopes, which would only be unsealed by research nurse after patient registration. Investigators were responsible for patient recruitment and the implementation of intervention allocation.

### Pretreatment workup and staging

All patients received the following pretreatment examinations and staging: neck, thorax, and abdomen plain and contrast-enhanced computed tomography (CT); esophagogastroduodenoscopy, with ultrasound endoscopy (EUS); and cervical ultrasonography. If indicated, bronchoscopy was performed to exclude tumor infiltration into the trachea or bronchial tree. Positron emission tomography and radionuclide bone imaging were optional.

### Neoadjuvant therapy

Patients were enrolled and randomly assigned (1:1) according to the enrollment order. Toripalimab was administered at a fixed dose of 240 mg every cycle. Paclitaxel was administered at a dose of 150–175 mg/m² every cycle, and cisplatin was administered at a dose of 70–75 mg/m². Neither the investigators nor the patients were masked to treatment allocation. The patients in the sequential group received paclitaxel and cisplatin on day 1 and toripalimab on day 3. The patients in the concurrent group received paclitaxel and cisplatin and toripalimab on day 1. The treatment cycle was 21 days in the concurrent group and 21 days in the sequential group (**eFigure 1 in Supplementary 1**). Radical surgery was performed 4–6 weeks after the second chemotherapy plus toripalimab.

### Surgery

McKeown esophagogastrectomy or thoracoscopic McKeown esophagogastrectomy was performed for thoracic esophageal cancerwith extended two-field lymphadenectomy.

### Follow-up

The surveillance tests will include abdominal and cervical color Doppler ultrasonography and chest CT scans. All of these examinations will be conducted in the outpatient department. The frequency of the visits will be every 3 months during the first 2 years, every 6 months during the third to fifth years, and every year after the fifth year.

### Pathological evaluation

The depth of tumor invasion, lymph node metastasis, and surgical margin were evaluated according to the American Joint Committee on Cancer Criteria for esophageal carcinoma (27). The extent of the residual tumors was divided into four categories: grade 0, no evidence of viable tumor cells; grade 1, single cells or rare small groups of cancer cells; grade 2, residual cancer cells with evident tumor regression, but more than single cells or rare small groups of cancer cells; and grade 3, extensive residual cancer without evident tumor regression (28). The postoperative pathological evaluation was carried out by two experienced pathologists. The expression of PD-L1 was examined using the Dako 22C3 antibody and counted based on a CPS.

### Outcomes

The primary endpoint of this study was to explore the influence of sequence of toripalimab and chemotherapy on pCR rate in locally advanced ESCC. The secondary endpoints were to explore the safety of chemotherapy combined with toripalimab as neoadjuvant treatment under different administration sequences, evaluating treatment-related adverse events (AEs), which were evaluated according to the National Cancer Institute Common Terminology Criteria for Adverse Events Version 5.0 (NCI-CTC AE 5.0) and investigating the influence of overall survival (OS) and disease-free survival (DFS); OS was defined as the time from Day 1 of preoperative treatment to death from any cause, and DFS was defined as the time from the date of R0 resection to disease recurrence or death from any cause.

### Statistical analysis

Based on the primary endpoint of pCR rate, the sample size was calculated using PASS 15.0 software. Preliminary data showed that the pCR rate was 7% in the concurrent group (chemotherapy combined with toripalimab administered synchronously, n=15) and 36% in the sequential group (toripalimab administered 3 days after chemotherapy, n=15). With a two-tailed significance level α=0.05, a test power of 80%, and an expected dropout rate of 20%, the calculation using the two-sample rate comparison test (two-tailed) showed that at least 70 patients (35 in each group) needed to be enrolled to achieve statistical significance. Statistical analysis was performed using SPSS 22.0 software: categorical variables were presented as frequencies (percentages), and continuous variables were presented as mean ± standard deviation; OS and DFS were calculated using the Kaplan-Meier method, and comparisons between groups were performed using the log-rank test; R0 resection rate, complication rate, and treatment-related mortality were analyzed using the χ² test or Fisher’s exact test.

## Results

### Patients

**Figure 1:**
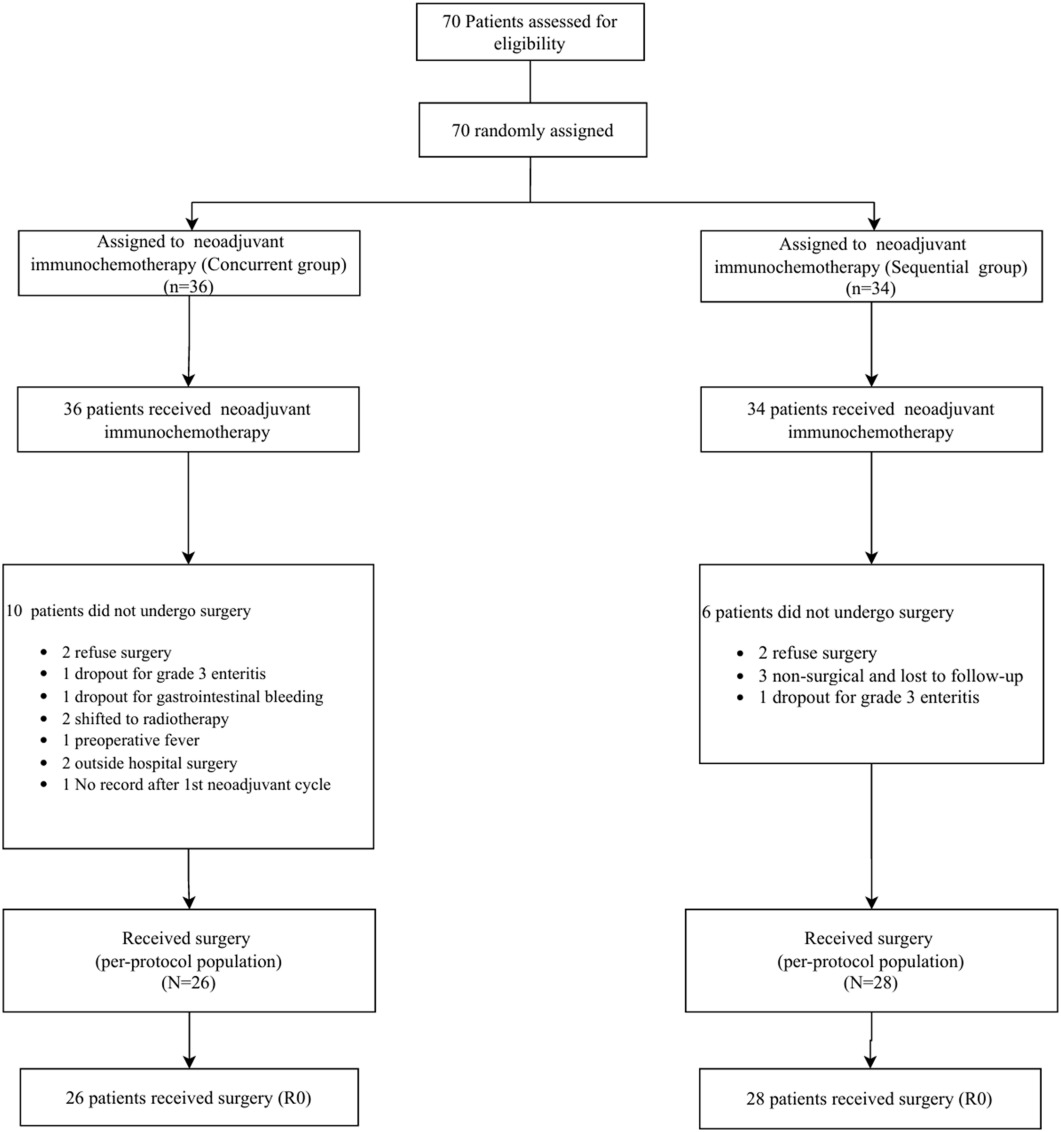
Study flow chart.

From July 2019 to June 2023, 70 patients were randomly assigned to the sequential group (n=34) and the concurrent group (n=36; **Figure 1**), The two groups were well balanced at baseline (**Table 1**).

**Table 1:** Baseline characteristics of different groups.

Variables	Total (n = 70)	Concurrent (n = 36)	Sequential (n = 34)	Statistic	*P*
Age, M (Q1, Q3)	66.00 (60.00, 70.00)	66.50 (62.50, 69.25)	65.00 (59.25, 70.00)	Z=-0.83	0.407
Gender, n(%)				χ²=0.23	0.632
male	56 (80.00)	28 (77.78)	28 (82.35)		
female	14 (20.00)	8 (22.22)	6 (17.65)		
ECOG PS, n(%)				χ²=0.00	0.967
0	41 (58.57)	21 (58.33)	20 (58.82)		
1	29 (41.43)	15 (41.67)	14 (41.18)		
Location, n(%)				–	0.590
upper	6 (8.57)	2 (5.56)	4 (11.76)		
middle	26 (37.14)	13 (36.11)	13 (38.24)		
lower	38 (54.29)	21 (58.33)	17 (50.00)		
Clinical T stage, n(%)				–	1.000
1/2	2 (2.86)	1 (2.78)	1 (2.94)		
3/4	68 (97.14)	35 (97.22)	33 (97.06)		
Clinical N stage, n(%)				χ²=0.02	0.888
0/1	52 (74.29)	27 (75.00)	25 (73.53)		
2/3	18 (25.71)	9 (25.00)	9 (26.47)		
Clinical stage, n(%)				χ²=5.58	0.061
II	16 (22.86)	7 (19.44)	9 (26.47)		
III	42 (60.00)	26 (72.22)	16 (47.06)		
IV	12 (17.14)	3 (8.33)	9 (26.47)		
PDL1-CPS (10), n(%)				χ²=5.18	0.075
Missing	13 (18.57)	5 (13.89)	8 (23.53)		
≥10	11 (19.30)	9 (29.03)	2 (7.69)		
<10	46 (80.70)	22 (70.97)	24 (92.31)		

Data are presented as n (%) or median (Q1, Q3).

n, number of patients; M, median; Q1, first quartile; Q3, third quartile; Z, Z value of Mann-Whitney U test; χ², Chi-square value; ECOG PS, Eastern Cooperative Oncology Group performance status; -, Fisher’s exact test; PDL1-CPS, programmed death ligand 1 combined positive score.

### Treatment compliance

The median NICT duration was 32 days [interquartile range (IQR), 29–36 days]. In the sequential group,28 of 34 patients (82.3%) completed the whole multimodality therapy. Reasons for not undergoing surgery [6 of 34 (17. 6%)] were patient refusal (n = 2), withdrawal due to grade 3 enteritis (n = 1), unsatisfactory performance status for surgery (n = 3); In the sequential group, 26 of 36 patients (82.3%) completed the whole multimodality therapy. Reasons for not undergoing surgery [10 of 36 (27. 7%)] were patient refusal(n = 2), withdrawal due to grade 3 enteritis (n = 1), gastrointestinal bleeding(n = 1), transfer to radiotherapy (n = 2), preoperative fever (n = 1), undergoing surgery at another hospital (n = 2), loss to follow-up after the first cycle of neoadjuvant treatment (n = 1). Sixty-five patients (92.8%) received two cycles of chemoimmunotherapy, while 5 patients (7.1%) received only one cycle of chemoimmunotherapy (**Supplementary eTable 2**).

### Safety

All patients experienced treatment-related adverse events (TRAEs), with the incidence of grade 3–5 TRAEs being 14.2% (10/70) (**Table 2**). Non-hematological toxicities were mainly characterized by Anorexia (91.42%, 64/70) and Loss of hair (77.1%, 54/70). Nausea (P = 0.028) and Diarrhea (P = 0.003), which had a higher incidence in the concurrent group. During treatment in the sequential group, 1 case of grade 3 Autoimmune disorder (enteritis) occurred, which resolved after treatment with glucocorticoids and plasma exchange. No deaths occurred within 30 days after surgery in either group. The definition of treatment-related death encompassed the period from initiation of neoadjuvant therapy through 90 days post-surgery. There were a total of 6 treatment-related deaths: 5 in the concurrent group (1 case each of Myocarditis(Immune-Related), Hyperglycemic Ketoacidosis, postoperative pneumonia, upper gastrointestinal bleeding, and post-radiotherapy esophageal fistula) and 1 in the sequential group (Gastric Hemorrhage). Regarding postoperative complications, 43 patients (79.6%) developed pulmonary infections, among which 3 progressed to sepsis (2 recovered after treatment with broad-spectrum antibiotics, and 1 died). A patient who died of sepsis had elevated troponin, suspected to be caused by immune-related myocarditis during sepsis. Surgery-related complications included 2 cases of anastomotic fistula (1 in the sequential group and 1 in the concurrent group), both of which healed successfully with conservative treatment without the need for reoperation. There was no significant difference in the incidence of postoperative complications between the two groups (P>0.05) (**Table 3**).

**Table 2:** Treatment-related adverse events.

Adverse event	Sequential group (n=34)		Concurrent group (n=36)		χ2	*P*
	Any grade	Grade 3/5	Any grade	Grade 3/5		
TRAES,N(%)	34(100.00)	4(11.76)	36(100.00)	6(16.66)	0.343	0.558^a^
Hyponatremia	6(16.66)	0	8(22.22)	1(2.77)	1.021	0.600^a^
Hypocalcemia	3(8.33)	0	5(13.88)	0	0.443	0.506 ^a^
Hyperglycemia	3(8.33)	0	4(11.11)	0	0.102	0.750 ^a^
Hypoalbuminemia	10(29.41)	0	9(25.00)	0	0.172	0.678
Hypokalemia	9(26.47)	2	7(19.44)	3(8.33)	1.258	0.533 ^a^
Anorexia	31(91.14)	0	33(91.66)	0	0.005	0.942
Hyperkalemia	1(2.94)	0	0	0	1.074	0.300
Rash	6(17.64)	1(2.94)	10(27.77)	0	2.686	0.261 ^a^
Hair Loss	25(73.52)	0	29(80.55)	0	0.490	0.484
Pruritus	9(26.47)	0	9(25.00)	1(2.77)	5.404	0.067
Fever	4(11.76)	0	3(8.33)	0	0.229	0.632 ^a^
Fatigue	18(52.94)	1(2.94)	18(50.00)	0	1.09	0.580
Pain	13(38.23)	2(5.88)	11(30.55)	1(2.77)	0.672	0.715 ^a^
Constipation	8(23.52)	0	15(41.66)	0	2.607	0.106
Nausea	6(16.66)	0	15(41.66)	0	4.804	0.028
Diarrhea	3(8.33)	0	14(38.88)	0	8.596	0.003^a^
Abdominal Distention	4(11.76)	0	6(16.66)	0	0.343	0.558 ^a^
Dry Mouth	13(38.23)	0	12(33.33)	0	0.183	0.669
Neuromuscular Toxicity	22(64.70)	0	24(66.66)	1(2.77)	0.030	0.863
Autoimmune disorder(enteritis)	1(2.94)	1(2.94)	0	0	1.074	0.300 ^a^

Data are presented as n (%).

n, Number of patients; TRAE, treatment-related adverse event. ^a^Fisher’s exact test.

**Table 3:** Postoperative complications.

Postoperative complications	Sequential group (n=28)	Concurrent group (n=26)	*P*
Pneumonia	22 (78.5%)	21 (80.7%)	0.841
Respiratory Failure	1 (3.6%)	0 (0.0%)	0.999 ^a^
Anastomotic fistula	1 (3.6%)	1 (3.8%)	0.998 ^a^
Pleural Effusion	6 (21.4%)	8 (30.7%)	0.434
Myocarditis (Immune-Related)	0	1 (3.8%)	0.295 ^a^
Pneumonitis (Immune-Related)	0	1 (3.8%)	0.295 ^a^
Hyperglycemia (Immune-Related)	0	1 (3.8%)	0.295 ^a^

Data are presented as n (%).

n, number of patients; a, Fisher’s exact test.

### Efficacy

**Figure 2:**
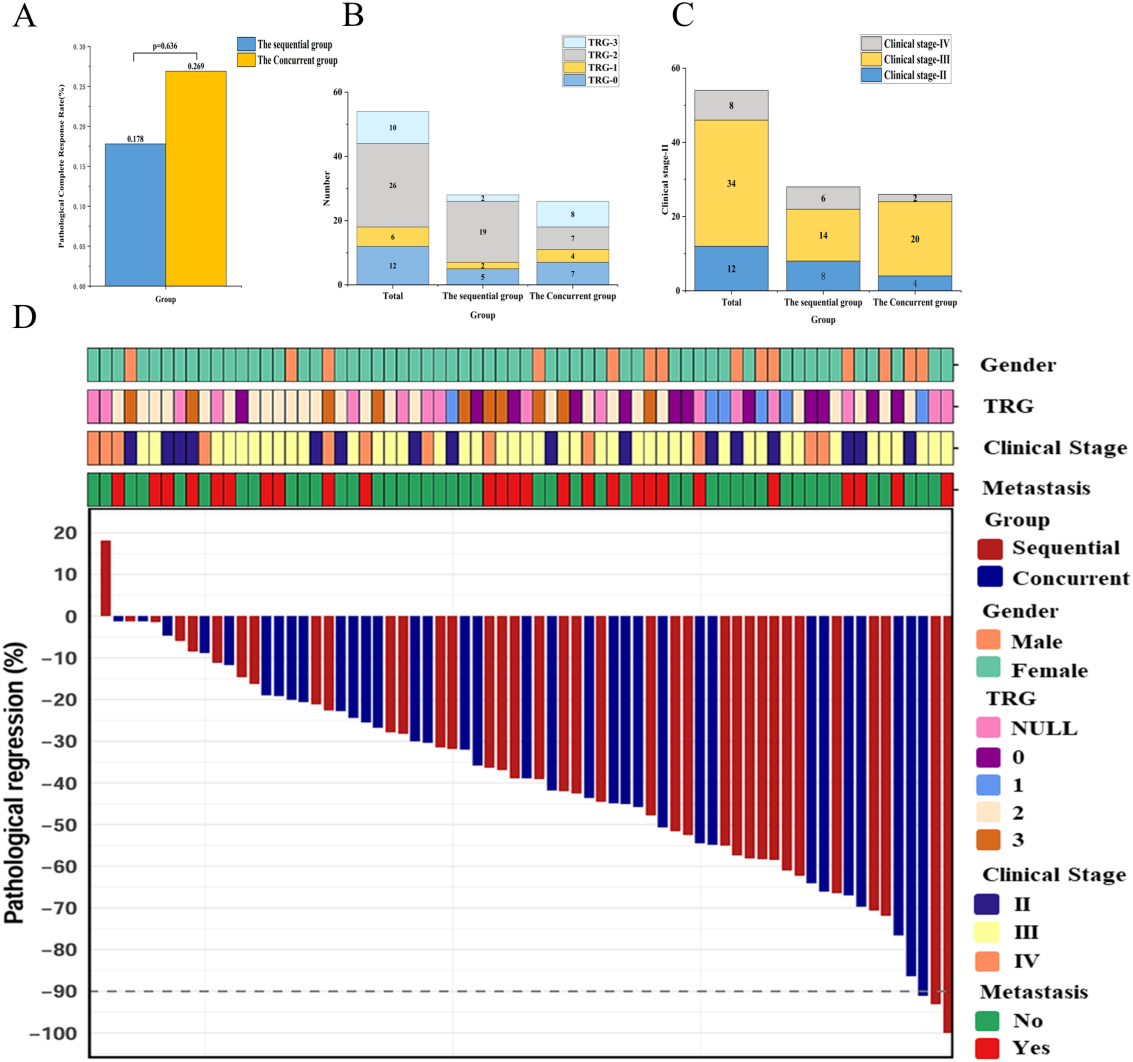
Tumor response to neoadjuvant treatment. **(A)**The pCR rates of the sequential group and the concurrent group; **(B)** TRG scores of the Sequential group and the concurrent group; **(C)** Clinical Staging of the Sequential group and the concurrent group; **(D)** The lower plot shows the pathological regression of the neoadjuvant treatments.

**Figure 3:**
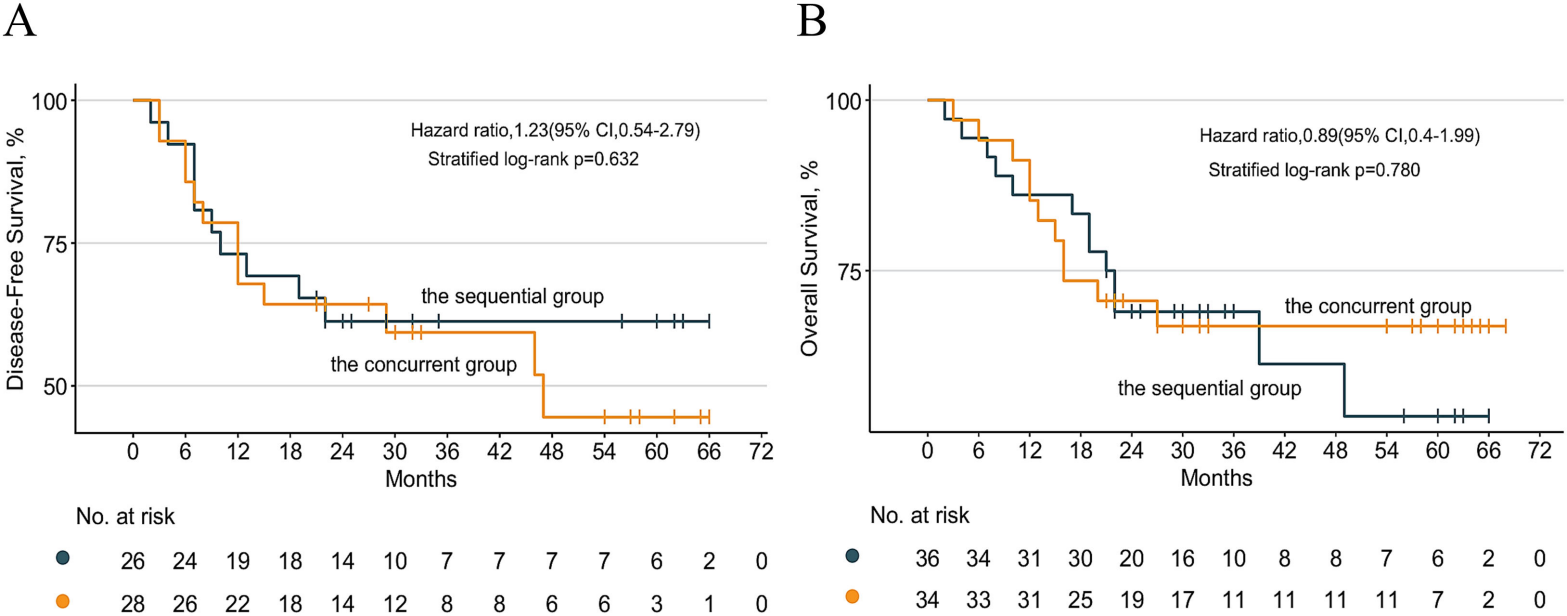
**(A)** DFS, disease-free survival; **(B)** OS, overall survival in the sequential vs concurrent groups.

Among the 70 enrolled patients, 54 underwent radical resection. All 54 patients (100%) achieved R0 resection. 12 (22.2%) achieved pathologic complete response (pCR), with no statistically significant intergroup difference [17.8% (sequential group) vs. 26.9% (concurrent group), P = 0.636; **Figure 2A**]. The wide confidence interval includes both null and potentially clinically meaningful effects, consistent with the study being underpowered to definitively exclude a true difference between strategies. The median number of lymph nodes dissected intraoperatively was 27.1 ± 12.4. Tumor regression grade (TRG) was categorized as follows: TRG 0 (12/54, 22.2%), TRG 1 (6/54, 11.1%), TRG 2 (26/54, 48.1%), and TRG 3 (10/54, 18.5%) (**Table 4**; **Figure 2B**). The clinical staging of patients who underwent surgery is shown in **Figure 2C**; the objective response rate (ORR) during neoadjuvant treatment was 57.1% (40/70; **Figure 2D**). For the entire cohort, the median follow-up duration was 27.0 months [interquartile range (IQR), 19.7–54.0]. Among the 70 patients, 12 (17.1%) developed recurrence and 6 (8.6%) Treatment-related deaths (**Supplementary eTable 1**). The 1-, 3-, and 5-year overall survival (OS) rates were 85.30%, 66.9%, and 66.9% in the concurrent group and 86.10%, 69.00%, and 53.7% in the sequential group (**Supplementary eTable 3**), respectively (P = 0.780; **Figure 3A**). For disease-free survival (DFS), the 1-, 3-, and 5-year rates were 67.90%, 59.30%, and 44.50% in the concurrent group and 73.10%, 61.30%, and 61.30% in the sequential group (**Supplementary eTable 4**), respectively (P = 0.632; **Figure 3B**).

**Table 4:** Surgical outcomes.

Characteristics	Total (n = 54)	Sequential group (n=28)	Concurrent group (n=26)	*P*
Operation time (min), mean ± SD	286.8 (± 67.7)	283.1 (± 68.0)	290.8 (± 68.6)	0.624
Number of lymph node dissection (n), mean ± SD	27.1 (± 12.4)	24.8 (± 11.9)	29.3 (± 12.7)	0.652
Surgical path, n (%)				0.645
Open	14 (25.9%)	8 (28.5%)	6 (23.0%)	
VATS	40 (74.0%)	20 (71.4%)	20 (76.9%)	
Blood loss (ml), mean ± SD	152.0 (± 101.3)	146.4 (± 107.0)	158.0 (± 96.4)	0.804
ypT stage, n (%)				0.028
T0-T1	18 (33.3%)	6 (21.4%)	13 (50%)	
T2-T3	35 (66.6%)	22 (78.5%)	13 (50%)	
ypN stage, n (%)				0.999
N0-N1	48 (88.8%)	25 (89.2%)	23 (88.4%)	
N2-N3	6 (11.2%)	3 (10.7%)	3 (11.5%)	
pCR	12 (22.2%)	5 (17.8%)	7 (26.9%)	0.636
TRG, n (%)				0.178
TRG 0/1	18 (33.3%)	7 (25.0%)	11 ( (42.3%)	
TRG 2/3	36 (66.7%)	21 (75.0%)	15 (57.6%)	
pCR ITT set (n=70)	12 (17.1%)	5 (14.7%)	7 (19.4%)	0.754

Data are presented as n (%) or mean ± SD.

n, number of patients; SD, standard deviation; VATS, video-assisted thoracoscopic surgery; ypT, pathological tumor stage after neoadjuvant therapy; ypN, pathological lymph node stage after neoadjuvant therapy; pCR, pathological complete response; TRG, tumor regression grade; ITT, intention-to-treat.

## Discussion

To our knowledge, the trial represents the first prospective, randomized, controlled phase II study designed to explore the “immune sensitive window” in ESCC through a direct head-to-head comparison of sequential versus concurrent neoadjuvant immunochemotherapy. Although neoadjuvant chemotherapy combined with PD-1 inhibitors has become a pivotal therapeutic strategy, the optimal sequencing of these two agents remains a subject of debate. Contrary to our initial hypothesis, the results demonstrated that delaying toripalimab administration to Day 3 did not yield a statistically significant improvement in pathological complete response (pCR) rates or survival outcomes compared to the standard concurrent regimen. However, the sequential strategy exhibited a numerically favorable safety signal, characterized by a markedly lower incidence of gastrointestinal toxicity and fatal adverse events. These exploratory findings generated the hypothesis that separation of agents might mitigate toxicity. This requires confirmation in adequately powered studies.

The primary efficacy endpoint revealed a pCR rate of 17.8% in the sequential group versus 26.9% in the concurrent group (26), a finding that diverged from a previous pilot study and recent data in other solid tumors (23). A prior pilot study of 30 patients had suggested a trend where in the sequential strategy might achieve a higher pCR rate (36% vs. 7%). This discrepancy likely stems from the small sample size of the pilot trial, where the concurrent group’s pCR rate of 7% was anomalously low compared to historical controls. Conversely, in the current study, the concurrent group’s pCR rate of 26.9% aligns closely with efficacy benchmarks reported in large-scale phase III immunochemotherapy trials for ESCC, there by validating the reliability of our control arm data (8, 29).

A critical theoretical basis of this study design was the capture of the chemotherapy-induced hypothesized immunological window The selection of a 2-day interval (administering immunotherapy on Day 3) was grounded in the positive clinical signals from the pilot study and a mechanistic understanding of chemotherapy-induced immune kinetics. Mechanistically, as elucidated by Huo et al., chemotherapy induces a transient phase of systemic immunosuppression immediately following administration. Their dynamic immune profiling showed that PD-1 expression on CD8+ T cells was downregulated on Days 1 and 2 but recovers and peaks on Day 3 (23). Theoretically, introducing PD-1 inhibitors during this time window allows for the avoidance of acute lymphotoxicity while synergizing with chemotherapy-induced immunogenic cell death (ICD) (30–32). However, the failure to replicate the efficacy benefits observed in NSCLC suggests that this “immune window” may be tumor-type specific, regimen-dependent or lack of sample size. The immunomodulatory temporal patterns induced by the paclitaxel-cisplatin regimen used in ESCC may differ from those of the pemetrexed-platinum regimen used in lung cancer. For ESCC, the immunosuppressive phase might be prolonged, or hypothesized immunological window may fall outside the Day 3 time point tested in this protocol.

Despite the lack of superior efficacy, the safety analysis provided compelling evidence supporting the sequential administration strategy. The concurrent group exhibited significantly higher incidences of grade 3–5 treatment-related adverse events, particularly nausea and diarrhea, and a higher number of treatment-related deaths (5 cases vs. 1 case). This observation supports the hypothesis that concurrent administration may exacerbate toxicity through an “additive effect,” where the acute emetogenic and cytotoxic effects of chemotherapy temporally overlap with the initial cytokine release or immune activation induced by PD-1 inhibitors. Previous studies have indicated that temporally separating chemotherapy and immunotherapy can facilitate better weight recovery and reduce side effects in animal models (19). In this study, the sequential approach likely mitigated this cumulative toxicity, potentially averting the severe immune-related adverse events (irAEs)—such as fatal myocarditis and hyperglycemic ketoacidosis—observed in the concurrent arm. If confirmed in larger trials, these observations might suggest that sequential administration could be considered for patients with poor performance status.

Several limitations of this study warrant consideration. First, study power and effect size uncertainty. The trial was powered on pilot data (n=15/group) showing a 29% pCR difference. This optimistic estimate likely left the study underpowered for realistic effect sizes. Moreover, pCR assessment was limited to surgical patients (n=54, 77%), further reducing effective sample size. Consequently, the non-significant pCR comparison cannot be interpreted as proof of equivalence between strategies. The definitive conclusions require an adequately powered phase III trial. Second, the pilot study coincided with the COVID-19 outbreak, leading to treatment delays or surgical postponements for some patients, which may have attenuated the tumor regression effects observed at that time. Crucially, in the sequential group of the current study, only two patients had a PD-L1 Combined Positive Score (CPS) ≥10, suggesting a population with relatively low sensitivity to immunotherapy, which may be a primary reason for the absence of positive efficacy results. Third, the specific biological mechanisms underlying the disparity in fatal irAEs between the two groups require further translational research to determine if concurrent administration triggers a distinct, more aggressive inflammatory pathway in ESCC. Finally, the baseline imbalances. Despite randomization, the sequential group exhibited a higher proportion of Stage IVa disease (26.5% versus 8.3%) and a lower proportion of Stage III disease (47.1% versus 72.2%) compared with the concurrent group. The imbalances might explain our failure to replicate the pilot study’s positive hint. The sequential group’s numerically lower pCR rate likely reflected baseline disease burden rather than inferiority of the sequencing strategy. This is the inherent limitation of small phase II trials. A phase III study with adequately powered was needed for the final conclusion.

In conclusion, although sequential administration of toripalimab on Day 3 did not improve pathological response rates compared to the concurrent regimen in locally advanced ESCC, it offered substantial benefits regarding safety and tolerability. The rationale of targeting the Day 3 immune recovery window, while effective in other tumor types, may require further optimization in the context of esophageal cancer. Nevertheless, the reduction in severe gastrointestinal toxicity and treatment-related mortality highlighted the sequential strategy as a potentially safer therapeutic option. Future phase III studies should focus on validating these safety signals and exploring biomarkers capable of identifying patient subgroups who might biologically benefit from immunochemotherapy sequencing.

## Conclusions

There was no significant difference in pCR between sequential and concurrent immunochemotherapy, but the concurrent strategy led to an increased risk of nausea/vomiting, diarrhea, and fatal irAEs.

## Data Availability

The original contributions presented in the study are included in the article/**Supplementary Material**. Further inquiries can be directed to the corresponding author.

## References

[1] Bray F Laversanne M Sung H Ferlay J Siegel RL Soerjomataram I . Global cancer statistics 2022: GLOBOCAN estimates of incidence and mortality worldwide for 36 cancers in 185 countries. CA Cancer J Clin. (2024) 74(3):229–63. doi: 10.3322/caac.21834. PMID: 38572751

[2] Han B Zheng R Zeng H Wang S Sun K Chen R . Cancer incidence and mortality in China, 2022. J Natl Cancer Cent. (2024) 4:47–53. doi: 10.1016/j.jncc.2024.01.006. PMID: 39036382 PMC11256708

[3] Kojima T Shah MA Muro K François E Adenis A Hsu CH . Randomized phase III KEYNOTE-181 study of pembrolizumab versus chemotherapy in advanced esophageal cancer. J Clin Oncol. (2020) 38:4138–48. doi: 10.1200/JCO.20.01888. PMID: 33026938

[4] Sun JM Shen L Shah MA Enzinger P Adenis A Doi T . Pembrolizumab plus chemotherapy versus chemotherapy alone for first-line treatment of advanced oesophageal cancer (KEYNOTE-590): a randomised, placebo-controlled, phase 3 study. Lancet. (2021) 398:759–71. doi: 10.1016/S0140-6736(21)01234-4. PMID: 34454674

[5] Luo H Lu J Bai Y Mao T Wang J Fan Q . Effect of camrelizumab vs placebo added to chemotherapy on survival and progression-free survival in patients with advanced or metastatic esophageal squamous cell carcinoma: The ESCORT-1st randomized clinical trial. JAMA. (2021) 326:916–25. doi: 10.1001/jama.2021.12836. PMID: 34519801 PMC8441593

[6] Lu Z Wang J Shu Y Liu L Kong L Yang L . Sintilimab versus placebo in combination with chemotherapy as first line treatment for locally advanced or metastatic oesophageal squamous cell carcinoma (ORIENT-15): multicentre, randomised, double blind, phase 3 trial. BMJ. (2022) 377:e068714. doi: 10.1136/bmj-2021-068714. PMID: 35440464 PMC9016493

[7] Xu J Kato K Raymond E Hubner RA Shu Y Pan Y . Tislelizumab plus chemotherapy versus placebo plus chemotherapy as first-line treatment for advanced or metastatic oesophageal squamous cell carcinoma (RATIONALE-306): a global, randomised, placebo-controlled, phase 3 study. Lancet Oncol. (2023) 24:483–95. doi: 10.1016/S1470-2045(23)00108-0. PMID: 37080222

[8] Wang ZX Cui C Yao J Zhang Y Li M Feng J . Toripalimab plus chemotherapy in treatment-naïve, advanced esophageal squamous cell carcinoma (JUPITER-06): A multi-center phase 3 trial. Cancer Cell. (2022) 40:277–288.e3. doi: 10.1016/j.ccell.2022.02.007. PMID: 35245446

[9] Wu M Huang Q Xie Y Wu X Ma H Zhang Y . Improvement of the anticancer efficacy of PD-1/PD-L1 blockade via combination therapy and PD-L1 regulation. J Hematol Oncol. (2022) 15:24. doi: 10.1186/s13045-022-01242-2. PMID: 35279217 PMC8917703

[10] Galluzzi L Vitale I Aaronson SA Abrams JM Adam D Agostinis P . Molecular mechanisms of cell death: recommendations of the Nomenclature Committee on Cell Death 2018. Cell Death Differ. (2018) 25:486–541. doi: 10.1038/s41418-017-0012-4. PMID: 29362479 PMC5864239

[11] Patel SA Minn AJ . Combination cancer therapy with immune checkpoint blockade: mechanisms and strategies. Immunity. (2018) 48:417–33. doi: 10.1016/j.immuni.2018.03.007. PMID: 29562193 PMC6948191

[12] Galluzzi L Vitale I Warren S Adjemian S Agostinis P Martinez AB . Consensus guidelines for the definition, detection and interpretation of immunogenic cell death. J Immunother Cancer. (2020) 8:e000337. doi: 10.1136/jitc-2019-000337. PMID: 32209603 PMC7064135

[13] Salas-Benito D Pérez-Gracia JL Ponz-Sarvisé M Rodriguez-Ruiz ME Martínez-Forero I Castañón E . Paradigms on immunotherapy combinations with chemotherapy. Cancer Discov. (2021) 11:1353–67. doi: 10.1158/2159-8290.CD-20-1312. PMID: 33712487

[14] Yao W Zhao X Gong Y Zhang M Zhang L Wu Q . Impact of the combined timing of PD-1/PD-L1 inhibitors and chemotherapy on the outcomes in patients with refractory lung cancer. ESMO Open. (2021) 6:100094. doi: 10.1016/j.esmoop.2021.100094. PMID: 33780892 PMC8041717

[15] Bezu L Sauvat A Humeau J Gomes-da-Silva LC Iribarren K Forveille S . eIF2α phosphorylation is pathognomonic for immunogenic cell death. Cell Death Differ. (2018) 25:1375–93. doi: 10.1038/s41418-017-0044-9. PMID: 29358668 PMC6113215

[16] Beyranvand Nejad E van der Sluis TC van Duikeren S van Duikeren S Yagita H Janssen GM . Tumor eradication by cisplatin is sustained by CD80/86-mediated costimulation of CD8+ T cells. Cancer Res. (2016) 76:6017–29. doi: 10.1158/0008-5472.CAN-16-0881. PMID: 27569212

[17] Bally APR Austin JW Boss JM . Genetic and epigenetic regulation of PD-1 expression. J Immunol. (2016) 196:2431–7. doi: 10.4049/jimmunol.1502643. PMID: 26945088 PMC4780223

[18] Voorwerk L Slagter M Horlings HM Sikorska K van de Vijver KK de Maaker M . Immune induction strategies in metastatic triple-negative breast cancer to enhance the sensitivity to PD-1 blockade: the TONIC trial. Nat Med. (2019) 25:920–8. doi: 10.1038/s41591-019-0432-4. PMID: 31086347

[19] Fu D Wu J Lai J Liu Y Zhou L Chen L . T cell recruitment triggered by optimal dose platinum compounds contributes to the therapeutic efficacy of sequential PD-1 blockade in a mouse model of colon cancer. Am J Cancer Res. (2020) 10:473–90. PMC7061747 32195021

[20] Weir GM Hrytsenko O Quinton T . Anti-PD-1 increases the clonality and activity of tumor infiltrating antigen specific T cells induced by a potent immune therapy consisting of vaccine and metronomic cyclophosphamide. J Immunother Cancer. (2016) 4:68. doi: 10.1186/s40425-016-0169-2 27777777 PMC5067905

[21] Messenheimer DJ Jensen SM Afentoulis ME Wegmann KW Feng Z Friedman DJ . Timing of PD-1 blockade is critical to effective combination immunotherapy with anti-OX40. Clin Cancer Res. (2017) 23:6165–77. doi: 10.1158/1078-0432.CCR-16-2677. PMID: 28855348 PMC5641261

[22] He X Du Y Wang Z Wang X Duan J Wan R . Upfront dose-reduced chemotherapy synergizes with immunotherapy to optimize chemoimmunotherapy in squamous cell lung carcinoma. J Immunother Cancer. (2020) 8:e000807. doi: 10.1136/jitc-2020-000807. PMID: 33115941 PMC7594539

[23] Huo Y Wang D Yang S Xu Y Qin G Zhao C . Optimal timing of anti-PD-1 antibody combined with chemotherapy administration in patients with NSCLC. J Immunother Cancer. (2024) 12:e009627. doi: 10.1136/jitc-2024-009627. PMID: 39706602 PMC11667274

[24] Gao L Lu J Zhang P Hong ZN Kang M . Toripalimab combined with docetaxel and cisplatin neoadjuvant therapy for locally advanced esophageal squamous cell carcinoma: a single-center, single-arm clinical trial (ESONICT-2). J Gastrointest Oncol. (2022) 13:478–87. doi: 10.21037/jgo-22-131. PMID: 35557591 PMC9086050

[25] Xu J Bai Y Xu N Li E Wang B Wang J . Tislelizumab plus chemotherapy as first-line treatment for advanced esophageal squamous cell carcinoma and gastric/gastroesophageal junction adenocarcinoma. Clin Cancer Res. (2020) 26:4542–50. doi: 10.1158/1078-0432.CCR-19-3561. PMID: 32561664

[26] Xing W Zhao L Zheng Y Liu B Liu X Li T . The sequence of chemotherapy and toripalimab might influence the efficacy of neoadjuvant chemoimmunotherapy in locally advanced esophageal squamous cell cancer-a phase II study. Front Immunol. (2021) 12:772450. doi: 10.3389/fimmu.2021.772450. PMID: 34938292 PMC8685246

[27] Rice TW Ishwaran H Ferguson MK Blackstone EH Goldstraw P . Cancer of the esophagus and esophagogastric junction: an eighth edition staging primer. J Thorac Oncol. (2017) 12:36–42. doi: 10.1016/j.jtho.2016.10.016. PMID: 27810391 PMC5591443

[28] Zhang X Jain D . Updates in staging and pathologic evaluation of esophageal carcinoma following neoadjuvant therapy. Ann N Y Acad Sci. (2020) 1482:163–76. doi: 10.1111/nyas.14462. PMID: 32892349

[29] Qin J Xue L Hao A Guo X Jiang T Ni Y . Neoadjuvant chemotherapy with or without camrelizumab in resectable esophageal squamous cell carcinoma: the randomized phase 3 ESCORT-NEO/NCCES01 trial. Nat Med. (2024) 30:2549–57. doi: 10.1038/s41591-024-03064-w. PMID: 38956195 PMC11405280

[30] Liu B Hu X Feng K Gao R Xue Z Zhang S . Temporal single-cell tracing reveals clonal revival and expansion of precursor exhausted T cells during anti-PD-1 therapy in lung cancer. Nat Cancer. (2021) 3:108–21. doi: 10.1038/s43018-021-00292-8 35121991

[31] Mazzaschi G Facchinetti F Missale G Canetti D Madeddu D Zecca A . The circulating pool of functionally competent NK and CD8+ cells predicts the outcome of anti-PD1 treatment in advanced NSCLC. Lung Cancer. (2019) 127:153–63. doi: 10.1016/j.lungcan.2018.11.038. PMID: 30642544

[32] Luoma AM Suo S Wang Y Gunasti L Porter CBM Nabilsi N . Tissue-resident memory and circulating T cells are early responders to pre-surgical cancer immunotherapy. Cell. (2022) 185:2918–2935.e29. doi: 10.1016/j.cell.2022.06.018. PMID: 35803260 PMC9508682

