# Supplementary material for: Sequential versus concurrent neoadjuvant immunochemotherapy in locally advanced esophageal squamous cell carcinoma: a randomized, controlled, open-label, phase 2 trial (HCHTOG1906)
Source: Front Immunol. 2026 May 7;17:1770662. doi: 10.3389/fimmu.2026.1770662 (PMC13189760; doi:10.3389/fimmu.2026.1770662)
Supplement: Supplementary file 1 [file DataSheet1.pdf]

Sequential Versus Concurrent Neoadjuvant Immunochemotherapy in Locally Advanced Esophageal Squamous Cell Carcinoma: A Randomized, Controlled, Open-Label, Phase 2 Trial (HCHTOG1906)

Principal investigators

Professor Quanli Gao

Department of Immunotherapy, Cancer Hospital Affiliated to Zhengzhou University and Henan Cancer Hospital, Zhengzhou, Henan 450008, P. R. China

E-mail: gaoquanli2015@126.com

Professor Qunxing Wen

Department of Thoracic Surgery, The Affiliated Cancer Hospital of Zhengzhou University&Henan Cancer Hospital, Zhengzhou, Henan 450008, P. R. China

E-mail: wenqunxingvip@126.com

List of abbreviations:

AE: Adverse Event
ALB: albumin
ALT: Alanine Transaminase
APTT: Activated Partial Thromboplastin Time
AST: Aspartate Transaminase
BNP: Brain Natriuretic Peptide
BUN: Blood Urea Nitrogen
Cr: Creatine
CR: Complete Response
CRF: Case Report Form
CRP: C-reactive protein
DBIL: Direct Bilirubin
DCR: Disease Control Rate
DFS: Disease-free Survival
ECOG: Eastern Cooperative Oncology Group
FAS: Full Analysis Set
Fib: Fibrinogen
GLU: Blood Glucose
HGB: Hemoglobin
HIV: Human Immunodeficiency Virus
INR: International Normalized Ratio
LDH: Lactic Dehydrogenase
LYMPH: Lymphocyte Count
NEUT: Neutrophil Differential Count
ORR: Objective Response Rate
OS: Overall survival
PD: Progressive Disease
PLT: Platelet Count
PR: Partial Response
PT: Prothrombin Time
RBC: Red Blood Cell Count
RECIST: Response Evaluation Criteria in Solid Tumors
SAE: Serious Adverse Event
SS: Safety Set
TBIL: Total Bilirubin
TP: Total Protein
TT: Thrombin Time
UA: Uric Acid
UNL: Upper Normal Limit
WBC: White Blood Cell Count

1.Introduction

Esophageal cancer remains a major malignant tumor threatening the health of Chinese residents. Epidemiological studies on esophageal cancer in China in 2013 showed that its incidence and mortality rates ranked 6th and 4th among all malignant tumors, respectively[1].

Currently, surgical treatment is still the main therapeutic approach for esophageal cancer. However, for patients with locally advanced esophageal cancer (Stage IIA-III), the 5-year survival rate of radical surgery alone is less than 40%, and most patients experience local recurrence or metastasis within 3 years after surgery2. Neoadjuvant chemotherapy helps reduce tumor stage, eliminate systemic micrometastases, and observe the tumor ’ s response to chemotherapy, thereby guiding postoperative treatment 3.

Surgery is one of the primary curative treatments for esophageal cancer: it can achieve a curative effect in the early stage, while comprehensive treatment centered on surgery can cure some patients with advanced disease. Approximately 90% of esophageal cancer patients in China have esophageal squamous cell carcinoma (ESCC). For patients with no distant metastasis at initial diagnosis, experienced thoracic surgeons first assess the resectability or potential resectability of the tumor. Preoperative chemotherapy and/or radiotherapy (neoadjuvant chemotherapy or radiotherapy) is then used to convert potentially resectable tumors into resectable ones, thereby improving the cure rate. Preoperative radiotherapy increases the difficulty of wound healing, and patients who undergo surgery after preoperative radiotherapy or concurrent chemoradiotherapy have a higher incidence of postoperative complications. Thus, preoperative neoadjuvant chemotherapy and the selection of neoadjuvant chemotherapy regimens have long been key research areas for experts in the field of esophageal cancer treatment, with no randomized controlled clinical trial results available to date. A retrospective study involving 228 ESCC patients who received neoadjuvant chemotherapy followed by R0 resection found that the neoadjuvant treatment regimen and pretreatment T stage were independent factors affecting postoperative disease-free survival (DFS). Patients treated with the paclitaxel-platinum dual-drug regimen had longer DFS and overall survival (OS).

The PD-1 molecule is typically expressed on the surface of activated T cells, B cells, monocytes, and NK cells and acts as an inhibitory molecule. It is activated upon binding to its ligands (PD-L1 or PD-L2), thereby inhibiting the activity of immune cells. PD-L1 is mainly expressed on the surface of tumor cells and can also be expressed in some host cells (such as myeloid cells, lymphocytes, epithelial cells, and antigen-presenting cells). The binding of PD-1 to PD[4]-L1 inhibits the proliferation and survival of cytotoxic T lymphocytes, induces the apoptosis of tumor-infiltrating lymphocytes (TILs), and promotes the differentiation of CD4+ T cells into regulatory T cells (Treg). Anti-PD-1 antibodies have shown clear clinical efficacy in advanced esophageal cancer; in lung cancer and nasopharyngeal carcinoma, chemotherapy combined with anti-PD-1 antibodies has demonstrated better clinical efficacy than conventional chemotherapy alone. However, data on anti-PD-1 antibodies combined with chemotherapy in early-stage ESCC remain scarce[5].

Toripalimab is a selectively humanized high-affinity anti-PD-1 monoclonal antibody of the IgG4 subtype, independently developed by Junshi Biosciences. It has shown favorable clinical efficacy in a variety of solid tumors, including melanoma and urothelial carcinoma[6], with an incidence and severity of adverse events similar to those of other marketed anti-PD-1 monoclonal antibodies.

Based on the positive signals and clear limitations of previous studies[7], we expanded this prospective, randomized phase II clinical trial to provide prospective evidence for optimizing the combination mode of neoadjuvant chemoimmunotherapy through a head-to-head comparison between sequential and concurrent strategies, thereby guiding future clinical practice and the design of larger-scale phase III trials.

Reference:

1. Du L, Li R, Ge M, et al. Incidence and mortality of thyroid cancer in China, 2008-2012. *Chin J Cancer Res*. 2019;31(1):144-151. doi:10.21147/j.issn.1000-9604.2019.01.09

2. Minashi K, Nihei K, Mizusawa J, et al. Efficacy of Endoscopic Resection and Selective Chemoradiotherapy for Stage I Esophageal Squamous Cell Carcinoma. *Gastroenterology*. 2019;157(2):382-390.e3. doi:10.1053/j.gastro.2019.04.017

3. Detecting Pathological Complete Response in Esophageal Cancer after Neoadjuvant Therapy Based on Imaging Techniques: A Diagnostic Systematic Review and Meta-Analysis - PubMed. Accessed October 29, 2025. https://pubmed.ncbi.nlm.nih.gov/30999111/

4. Takeuchi M, Kawakubo H, Mayanagi S, et al. Influence of Neoadjuvant Therapy on Poor Long-Term Outcomes of Postoperative Complications in Patients with Esophageal Squamous Cell Carcinoma: A Retrospective Cohort Study. *Ann Surg Oncol*. 2019;26(7):2081-2089. doi:10.1245/s10434-019-07312-z

5. Zhang X, Jia J, Lu M, et al. Nimotuzumab Plus Paclitaxel and Cisplatin as a 1st-Line Treatment for Esophageal Cancer: Long Term Follow-up of a Phase II Study. *Journal of Cancer*. 2019;10(6):1409-1416. doi:10.7150/jca.28659

6. Tang B, Yan X, Sheng X, et al. Safety and clinical activity with an anti-PD-1 antibody JS001 in advanced melanoma or urologic cancer patients. *J Hematol Oncol*. 2019;12(1):7. doi:10.1186/s13045-018-0693-2

7. Xing W, Zhao L, Zheng Y, et al. The Sequence of Chemotherapy and Toripalimab Might Influence the Efficacy of Neoadjuvant Chemoimmunotherapy in Locally Advanced Esophageal Squamous Cell Cancer—A Phase II Study. *Front Immunol*. 2021;12:772450. doi:10.3389/fimmu.2021.772450

2. Study objectives

2.1 Primary objective

- To explore the influence of sequence of toripalimab and chemotherapy on pCR rate

2.2 Secondary objective

- To explore the safety of chemotherapy combined with toripalimab as neoadjuvant treatment under different administration sequences
- To explore the evaluating treatment-related adverse events (AEs)
- To investigate the influence of overall survival (OS) and disease-free survival (DFS)

3. Study plan and procedures

3.1 Overall study design

The trial is a single-center, single-arm, open-label Phase II clinical study to evaluate the differences in efficacy and safety between sequential and concurrent administration of neoadjuvant chemotherapy (paclitaxel/cisplatin) combined with PD-1 inhibitor (toripalimab) in patients with resectable locally advanced ESCC.

Approximately 90% of esophageal cancer patients in China have ESCC, so this study mainly enrolls patients with resectable or potentially resectable ESCC. A total of 70 patients are planned to be enrolled and divided into two groups. The dose of toripalimab is fixed at 240mg (referenced from its previous clinical study recommendations), with a treatment cycle of every 3 weeks. The specific administration methods are as follows:

The Concurrent Group (36 patients)

- Toripalimab: 240mg, administered on Day 1
- Paclitaxel: 175mg/m², administered on Day 1
- Cisplatin: 75mg/m², administered on Day 1

Treatment is given every 3 weeks as one cycle. Efficacy will be evaluated after 2 cycles, followed by surgical resection.

The Sequential group(34 patients)

- Paclitaxel: 175mg/m², administered on Day 1
- Cisplatin: 75mg/m², administered on Day 1
- Toripalimab: 240mg, administered on Day 3

Treatment is given every 3 weeks as one cycle. Efficacy will be evaluated after 2 cycles, followed by surgical resection.

Adverse events will be closely observed during treatment, and detailed records will be made in the adverse event report form. Before enrollment: Contrast-enhanced CT and MR examinations will be performed to assess tumor burden and determine tumor staging. After 2 cycles of treatment: Preoperative CT and MR examinations will

be conducted to evaluate efficacy; postoperative pathological examination will be used for pathological efficacy assessment. Patients will undergo reexaminations every 12 weeks within 2 years after surgery, and every 24 weeks thereafter. Patient enrollment is planned to be completed within 1 year, and follow-up will continue for 3 years after the last patient is enrolled.

The study design is shown in Figure 1 and Table 1.

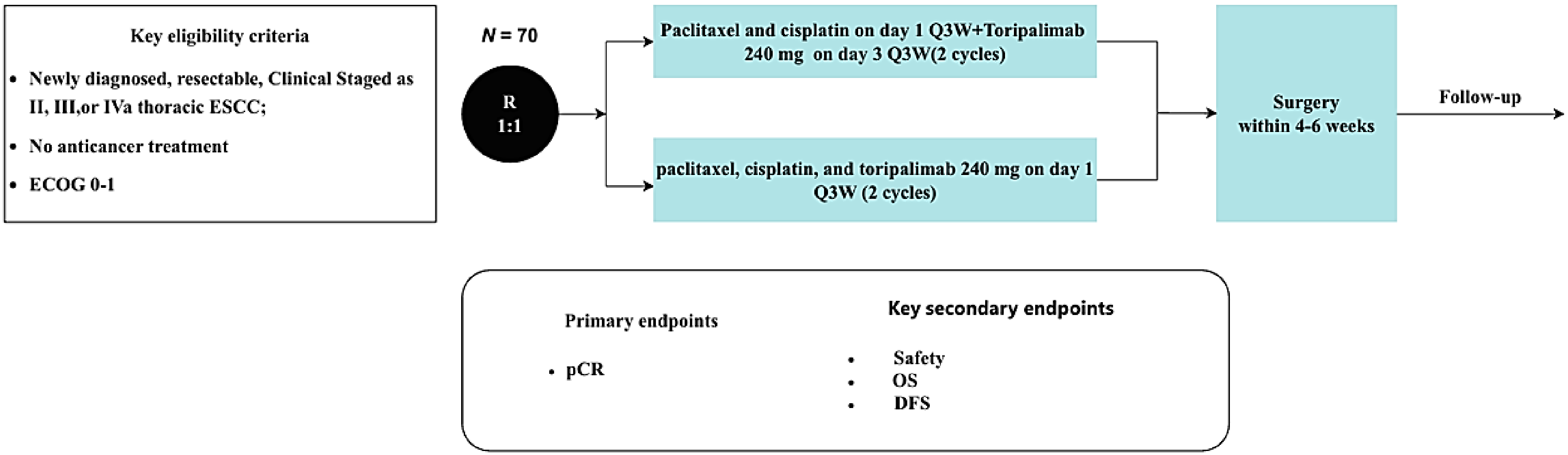

Figure 1. Study design of the HCHTOG1906 trial.

	Screening	Treatment					Safety	Follow up [9]
		Cycle 1	Cycle 2	Surgery	Subsequent Cycles	Post-Treatment Visit		
**Evaluation**	**-14~0 days**							
Written informed consent	×							
Demographic Data	×							
Medical History Data	×							
Vital Signs	×	×	×	×	×	×	×	
Physical Examination	×	×	×	×	×	×	×	
Eligibility	×							
Complete Blood Count (CBC)[1]	×		×	×	×	×	×	
Urinalysis[2]	×		×	×	×	×	×	
Blood Biochemistry Tests [3]	×		×	×	×	×	×	
Serum Pregnancy Test	×				×		×	
Coagulation Function Tests[4]	×		×	×	×	×	×	
Thyroid Function Tests[5]	×		×	×	×	×	×	
Immune Function Tests [6]	×		×	×	×			
Infectious Disease Screening[7]	×							
12-Lead Electrocardiogram	×		×	×	×	×	×	
Adverse Events (AEs)[8]	×			×	×	×	×	
Concomitant Medications		×	×	×	×	×	×	

Table1. Schedule of study procedures

NOTE 1. Complete Blood Count (CBC)

White Blood Cell Count (WBC), Red Blood Cell Count (RBC), Hemoglobin (Hb), Platelet Count (PLT), Neutrophil Count (NEUT), Lymphocyte Count (LYMPH)

2. Urinalysis

Protein, Glucose, Ketone Bodies, Leukocytes, Red Blood Cells, Occult Blood, pH Value

3. Blood Biochemistry Tests

Alanine Aminotransferase (ALT), Aspartate Aminotransferase (AST), Alkaline Phosphatase (ALP), Gamma-Glutamyl Transferase (GGT), Total Protein (TP), Albumin (ALB), Total Bilirubin (TBIL), Direct Bilirubin (DBIL), Blood Urea Nitrogen (BUN), Serum Creatinine (Scr), Uric Acid (UA), Total Bile Acid (TBA), Potassium (K^+), Sodium (Na^+), Chloride (Cl^-), Calcium (Ca^{2+}), Magnesium (Mg^{2+}), Lactate Dehydrogenase (LDH), α-Hydroxybutyrate Dehydrogenase (α-HBDH), Creatine Kinase (CK), Creatine Kinase Isoenzyme (CK-MB)

4. Coagulation Function Tests

Prothrombin Time (PT), Activated Partial Thromboplastin Time (APTT), Thrombin Time (TT), Fibrinogen (FIB), D-Dimer

5. Thyroid Function Tests

Free Triiodothyronine (FT3), Free Thyroxine (FT4), Thyroid-Stimulating Hormone (TSH)

6. Immune Function Tests

Helper T Cells/Suppressor T Cells (Th/Ts) Ratio, Regulatory T Cells (Tregs), PD-1$^+$ Cell Proportion in Peripheral Blood Mononuclear Cells (PBMCs), PD-1$^+$ Cell Proportion in CD3$^+$ Cells, PD-1$^+$ Cell Proportion in CD4$^+$ Cells, PD-1$^+$ Cell Proportion in CD8$^+$ Cells, Absolute Count of Total Lymphocytes, Absolute Count of T Lymphocytes, Absolute Count of Natural Killer (NK) Cells, Absolute Count of B Cells, Absolute Count of CD3$^+$CD4$^+$ Cells, Absolute Count of CD3$^+$CD8$^+$ Cells

7. Infectious Disease Screening

Hepatitis B Surface Antigen (HBsAg), Hepatitis C Virus Antibody (HCV-Ab), Human Immunodeficiency Virus Antibody (HIV-Ab), Treponema Pallidum Antibody (TP-Ab)

8. Tumor Assessment

Tumor evaluation will be performed in accordance with the Response Evaluation Criteria in Solid Tumors (RECIST) Version 1.1 and Immune RECIST (imRECIST) Criteria. The imaging method (CT or MRI) will be determined by the investigator, but the evaluation method, equipment and technical parameters must remain consistent throughout the study.

9. Regular Follow-up (Every 6 Weeks)

The tested indicators include: Thyroid Function (Free Triiodothyronine (FT3), Free Thyroxine (FT4), Thyroid-Stimulating Hormone (TSH)), Myocardial Enzyme Profile (Lactate Dehydrogenase (LDH), α-Hydroxybutyrate Dehydrogenase (α-HBDH), Creatine Kinase (CK), Creatine Kinase Isoenzyme (CK-MB)), Serum Amylase, Lipase, Brain Natriuretic Peptide (BNP), C-Reactive Protein (CRP)

4. Patient selection

4.1 Inclusion criteria

(I) Aged ≥18 years and ≤70 years, regardless of gender;
(II) Pathologically confirmed thoracic esophageal squamous cell carcinoma (ESCC);
(III) No distant metastasis confirmed by imaging examinations, and esophageal cancer deemed resectable or potentially resectable by thoracic surgery specialists after consultation;
(IV) Eastern Cooperative Oncology Group (ECOG) performance status score of 0–1;
(V) Clinical Staged as II, III, or IVa according to the 8th edition of the American Joint Committee on Cancer (AJCC) Cancer Staging Manual;
(VI) Sufficient organ function, defined as: ① No need for growth factor or blood component support within 2 weeks before enrollment; ② Cardiac function: No heart disease or coronary heart disease, with a cardiac function grade of 1－2; ③ Hepatic function: Total bilirubin (TBIL) ≤2 times the upper limit of normal (ULN), aspartate aminotransferase (AST) ≤2.5 ULN, alanine aminotransferase (ALT) ≤2.5 ULN; ④ Renal function: Creatinine (Cr) ≤ 1.25 ULN;
(VII) Normal blood pressure, or well-controlled blood pressure within the normal range using antihypertensive drugs for patients with hypertension;
(VIII) Fasting blood glucose ≤8 mmol/L controlled by hypoglycemic drugs for patients with diabetes;
(IX) No other severe diseases that conflict with this protocol (e.g., autoimmune diseases, immunodeficiency, organ transplantation);
(X) No history of other malignant tumors;
(XI) Women of childbearing age must have a negative serum pregnancy test within 7 days before enrollment, and all subjects of childbearing age must use appropriate contraceptive measures during the trial and within 6 months after the trial.

4.2 Exclusion criteria

(I) Pregnant or lactating women;
(II) History of severe infectious diseases within 4 weeks before enrollment;
(III) Patients with bronchial asthma requiring intermittent use of bronchodilators or medical intervention;
(IV) Use of immunosuppressants due to comorbid diseases before enrollment, with a dosage equivalent to ≥10 mg/day of oral prednisone for more than 2 consecutive weeks;
(V) Clinically significant cardiovascular and cerebrovascular diseases, including but not limited to severe acute myocardial infarction, unstable or severe angina pectoris, coronary artery bypass grafting, congestive heart failure, ventricular arrhythmias requiring medical intervention, or left ventricular ejection fraction (LVEF) <50% within 6 months before enrollment;
(VI) Severe allergic diathesis;
(VII) Severe mental disorders;
(VIII) Abnormal coagulation function (prothrombin time [PT] >16 s, activated partial thromboplastin time [APTT] >53 s, thrombin time [TT] >21 s, fibrinogen

[Fib] <1.5 g/L), bleeding tendency, or ongoing thrombolytic or anticoagulant therapy;Past or current pulmonary fibrosis, interstitial pneumonia, pneumoconiosis, radiation pneumonitis, severe impairment of pulmonary function, or other similar pulmonary conditions;

4.3 Discontinuation Criteria

During the study, if investigators judge that patients can no longer benefit from the investigational study, or if intolerable adverse effects or adverse effects that may cause serious consequences occur, investigators will determine whether to continue the combination therapy.

Reasons for Withdrawal

1. Subjects or their legal representatives withdraw informed consent;
2. Investigators determine that study participation should be terminated in the best interest of subjects;
3. Inability to continue investigational treatment due to adverse events (AEs);
4. Evidence showing that subjects cannot benefit from the investigational treatment;
5. Investigators and/or sponsors determine that subjects have seriously violated the study protocol (e.g., receiving other anti-tumor drug treatments);
6. Subjects are lost to follow-up.

Investigators must document the reason for withdrawal in the Case Report Form (CRF). They should attempt to contact the patient to complete all feasible assessment items, fill out the End-of-Treatment Follow-up Form, and record the time of the last medication administration if possible.For subjects who withdraw due to adverse events, and follow-up confirms that the adverse events are related to the study drug, such cases must be documented in the CRF and reported to the sponsor.

All existing study-related toxicities and Serious Adverse Events (SAEs) at the time of withdrawal must be followed up until resolution, unless in the investigator’s judgment, the condition cannot be resolved due to the patient’s underlying disease.

4.4 Withdrawal (Dropout) Criteria

Definition of Withdrawal

All patients who have signed the Informed Consent Form (ICF), passed the screening, and been enrolled in the study will be considered dropout cases if they fail to complete the full-course observation of the clinical study, regardless of the time or reason for withdrawal.

5. Patient enrollment and randomization

Patients were randomly assigned at a 1:1 ratio and allocated to the sequential group or concurrent group according to the enrollment sequence. The random assignment protocol was generated by computer at the Central Clinical Trial Center of Henan Cancer Hospital, using a simple randomization method. The assignments were placed in sealed envelopes, labeled by stratum, which would only be unsealed after patient registration. Central investigators were responsible for patient recruitment and the implementation of intervention allocation.

6. Treatment

Patients were enrolled and randomly assigned (1:1) according to the enrollment order. Toripalimab was administered at a fixed dose of 240 mg every cycle. Paclitaxel was administered at a dose of 150–175 mg/m² every cycle, and cisplatin was administered at a dose of 70–75 mg/m². Neither the investigators nor the patients were masked to treatment allocation. The patients in the sequential group received paclitaxel and cisplatin on day 1 and toripalimab on day 3. The patients in the concurrent group received paclitaxel and cisplatin and toripalimab on day 1. The treatment cycle was 21 days in the concurrent group and 21 days in the sequential group.

6.1 Treatment Drugs

6.11 Toripalimab

- Specification: 240mg/vial
- Manufacturer: Junshi Biosciences Co., Ltd.
- Administration: Intravenous injection, once every 3 weeks
- Adverse Reactions: In Phase I and II clinical studies of toripalimab, common adverse reactions included hypothyroidism, pruritus, elevated transaminases, elevated bilirubin, fever, and diarrhea. Other rare adverse reactions included neutropenia, thrombocytopenia, elevated troponin, and exacerbation of chronic lung disease. No treatment-related deaths were reported in clinical studies of toripalimab monotherapy.

6.12 Paclitaxel

- Ingredient: Paclitaxel
- Form: Injection
- Specification: 30mg/vial
- Manufacturer: Shenzhen Wanle Pharmaceutical Co., Ltd.
- Administration: 175mg/m², once every 3 weeks
- Adverse Reactions: Common adverse reactions of paclitaxel include alopecia, peripheral neurotoxicity, fatigue, cytopenia, elevated transaminases, and allergic reactions.

6.13 Cisplatin

- Ingredient: Cisplatin
- Form: Cisplatin Injection
- Specification: 30mg/vial
- Manufacturer: Jiangsu Hansoh Pharmaceutical Co., Ltd.
- Administration: 75mg/m², administered on Day 1
- Adverse Reactions: Common adverse reactions of cisplatin include nausea, vomiting, elevated serum creatinine, hearing loss, fatigue, hypoproteinemia, cytopenia, elevated transaminases, and elevated blood urea nitrogen.

6.2 Treatment Period

(1) First Cycle of Treatment (Day 1)

The Concurrent Group (36 patients)

- Toripalimab: 240mg, administered on Day 1
- Paclitaxel: 175mg/m², administered on Day 1
- Cisplatin: 75mg/m², administered on Day 1

The Sequential group(34 patients)

- Paclitaxel: 175mg/m^2, administered on Day 1
- Cisplatin: 75mg/m^2, administered on Day 1
- Toripalimab: 240mg, administered on Day 3

Treatment is repeated every 3 weeks.

(2) Second Cycle (Day 22±3)

- Vital sign measurement;
- Physical examination;
- Laboratory tests:
- Complete Blood Count (CBC): White blood cell count, red blood cell count, hemoglobin, platelet count, neutrophil count, lymphocyte count;
- Blood Biochemistry Tests: Serum electrolytes (potassium, sodium, chloride, calcium, magnesium, phosphorus), alanine aminotransferase (ALT), aspartate aminotransferase (AST), alkaline phosphatase (ALP), gamma-glutamyl transferase (GGT), total protein (TP), albumin (ALB), total bilirubin (TBIL), direct bilirubin (DBIL), blood urea nitrogen (BUN), serum creatinine (Scr), uric acid (UA), total bile acid (TBA), blood glucose, lactate dehydrogenase (LDH), α-hydroxybutyrate dehydrogenase (α-HBDH), creatine kinase (CK), creatine kinase isoenzyme (CK-MB);
- Urinalysis: Protein, glucose, ketone bodies, leukocytes, red blood cells, occult blood, pH value;
- Immune Function Tests: Helper T cells/Suppressor T cells (Th/Ts) ratio, regulatory T cells (Tregs), proportion of PD-1^+ cells in peripheral blood mononuclear cells (PBMCs), proportion of PD-1^+ cells in $CD3^+$ cells, proportion of PD-1^+ cells in $CD4^+$ cells, proportion of PD-1^+ cells in $CD8^+$ cells, absolute count of total lymphocytes, absolute count of T lymphocytes, absolute count of natural killer (NK) cells, absolute count of B cells, absolute count of $CD3^+CD4^+$ cells, absolute count of $CD3^+CD8^+$ cells;
- Thyroid Function Tests: Free triiodothyronine (FT3), free thyroxine (FT4), thyroid-stimulating hormone (TSH);
- Adverse event (AE) recording;
- Concomitant medication recording.

Medication Administration

The Concurrent Group (36 patients)

- Toripalimab: 240mg, administered on Day 22
- Paclitaxel: 175mg/m^2, administered on Day 22
- Cisplatin: 75mg/m^2, administered on Day 22-24

The Sequential group(34 patients)

- Paclitaxel: 175mg/m^2, administered on Day 22
- Cisplatin: 75mg/m^2, administered on Day 22
- Toripalimab: 240mg, administered on Day 25

Treatment is repeated every 3 weeks.

(3) Surgical Treatment (Day 43±7)

- Vital sign measurement;

- Physical examination;
- Laboratory tests:
- Complete Blood Count (CBC): White blood cell count, red blood cell count, hemoglobin, platelet count, neutrophil count, lymphocyte count;
- Blood Biochemistry Tests: Serum electrolytes (potassium, sodium, chloride, calcium, magnesium, phosphorus), alanine aminotransferase (ALT), aspartate aminotransferase (AST), alkaline phosphatase (ALP), gamma-glutamyl transferase (GGT), total protein (TP), albumin (ALB), total bilirubin (TBIL), direct bilirubin (DBIL), blood urea nitrogen (BUN), serum creatinine (Scr), uric acid (UA), total bile acid (TBA), blood glucose, lactate dehydrogenase (LDH), α-hydroxybutyrate dehydrogenase (α-HBDH), creatine kinase (CK), creatine kinase isoenzyme (CK-MB);
- Urinalysis: Protein, glucose, ketone bodies, leukocytes, red blood cells, occult blood, pH value;
- Immune Function Tests: Helper T cells/Suppressor T cells (Th/Ts) ratio, regulatory T cells (Tregs), proportion of $PD-1^+$ cells in peripheral blood mononuclear cells (PBMCs), proportion of $PD-1^+$ cells in $CD3^+$ cells, proportion of $PD-1^+$ cells in $CD4^+$ cells, proportion of $PD-1^+$ cells in $CD8^+$ cells, absolute count of total lymphocytes, absolute count of T lymphocytes, absolute count of natural killer (NK) cells, absolute count of B cells, absolute count of $CD3^+CD4^+$ cells, absolute count of $CD3^+CD8^+$ cells;
- Thyroid Function Tests: Free triiodothyronine (FT3), free thyroxine (FT4), thyroid-stimulating hormone (TSH);
- Contrast-enhanced CT of the chest, abdomen, and pelvis, plain and dynamic contrast-enhanced esophageal MRI, and/or plain and dynamic contrast-enhanced brain MRI;
- Adverse event (AE) recording;
- Concomitant medication recording.

Vital sign measurement, physical examination, and laboratory tests must be performed before each medication administration. Meanwhile, adverse events and concomitant medications experienced by subjects will be recorded. Postoperative adjuvant chemotherapy (if required) will be determined based on postoperative pathological findings; if adjuvant chemotherapy is needed, 2 cycles of paclitaxel combined with nedaplatin chemotherapy will be administered postoperatively. Imaging assessments will be conducted every 12 weeks for the first 2 years, and every 24 weeks thereafter.

If a subject discontinues treatment early due to adverse events, subject/guardian request, or other reasons, physical examination, laboratory tests, ECG, pulmonary function test, and imaging examinations will be performed at the time of discontinuation. Additionally, the Case Report Form (CRF) will be completed, adverse events will be recorded, and the reason for discontinuation will be described in detail.

7. Data collection

7.1 Data collection at enrollment

The following assessments and procedures should be performed within 14 days prior to randomization.

- Written informed consent for the study and assignment of a patient identification number

- Verify eligibility criteria
- Physical examination to assess all conditions that are current and ongoing
- WHO performance status
- Hematology, clinical chemistry
- 12-lead ECG recording
- Chest CT with contrast, Barium swallow, and Abdomen CT/MRI/ultrasound

7.2 Follow-up

The follow-up frequency was as follows: once every 3 months for the first 2 years after treatment, once every 6 months from the 3rd to the 5th year, and once a year after 5 years. Any disease and survival status and adverse events must be followed-up and noted in CRFs. Chest CT scan with contrast, abdomen CT/MRI/ultrasound and barium swallow should be processed as routine and endoscopy when necessary. Investigators will review all the results of the examinations above and make disease assessment.

7.3 Efficacy and safety data collection

The primary endpoint of this study was to explore the influence of sequence of toripalimab and chemotherapy on pCR rate in locally advanced ESCC. The secondary endpoints were to explore the safety of chemotherapy combined with toripalimab as neoadjuvant treatment under different administration sequences, evaluating treatment-related adverse events (AEs) , which were evaluated according to the National Cancer Institute Common Terminology Criteria for Adverse Events Version 5.0 (NCI-CTC AE 5.0) and investigating the influence of overall survival (OS) and disease-free survival (DFS); overall survival (OS) was defined as the time from Day 1 of preoperative treatment to death from any cause, and disease-free survival (DFS) was defined as the time from the date of R0 resection to disease recurrence or death from any cause.

8. Statistical methods and sample size determination

8.1 Description of analysis sets

The statistical analysis of pathological complete response (pCR) rate, the safety，overall survival (OS), and disease-free survival (DFS) will include all randomized patients and will compare the treatment arms on the basis of randomized treatment, regardless of the treatment actually received.All patients who received at least one cycle of randomized study drug will be included in the safety population. Throughout the safety results sections, patients with erroneous treatment allocation (e.g., those randomized to one group but actually receiving treatment of another group) will be accounted for in the actual treatment arm.

8.2 Methods of statistical analyses

The median OS and DFS will be estimated using the Kaplan-Meier method, and the log-rank test will be used to compare survival outcomes among treatment arms.
Adverse events (AEs) will be summarized by patient, and the number of patients experiencing each AE will be tabulated by treatment arm and graded according to the National Cancer Institute Common Terminology Criteria for Adverse Events Version 5.0 (NCI-CTC AE 5.0). Categorical variables will be presented as frequencies (percentages), and continuous variables will be presented as mean ± standard deviation. The χ^2 test or Fisher's exact test will be used to analyze R0 resection rate, complication rate, and treatment-related mortality.

8.3 Determination of the sample size

The sample size for this study was calculated based on the primary endpoint of pCR rate using PASS 15.0 software. Preliminary data showed that the pCR rate was 7% in the concurrent group (chemotherapy combined with toripalimab administered synchronously, n=15) and 36% in the sequential group (toripalimab administered 3 days after chemotherapy, n=15). With a two-tailed significance level $\alpha=0.05$, a test power of 80%, and an expected dropout rate of 20%, the two-sample rate comparison test (two-tailed) indicated that at least 70 patients (35 in each group) needed to be enrolled to achieve statistical significance. The total sample size was determined to be 70, considering the above parameters.

9. Ethics and dissemination

The study will be performed in accordance with ethical principles that have their origin in the Declaration of Helsinki and are consistent with Good Clinical Practice (GCP).This trial has been approved by the Ethics Committee of Henan Cancer Hospital. Written informed consent will be obtained from all participants. Serious adverse events will be reported to the safety desk of the trial, the Data and Safety Monitoring Board and trial sites.
