# Supplementary material for: Sequential versus concurrent neoadjuvant immunochemotherapy in locally advanced esophageal squamous cell carcinoma: a randomized, controlled, open-label, phase 2 trial (HCHTOG1906)
Source: Front Immunol. 2026 May 7;17:1770662. doi: 10.3389/fimmu.2026.1770662 (PMC13189760; doi:10.3389/fimmu.2026.1770662)
Supplement: Supplementary file 2 [file DataSheet2.docx]

**eFigure 1.** Study Design Abbreviations: ECOG, Eastern Cooperative Oncology Group; ESCC, esophageal squamous cell carcinoma; DFS, disease-free survival; OS, overall survival; pCR, pathological complete response; Q, quaque; R, random; T, tumor; N, node; W, week.

Graphical representation of the prospective, randomized, Controlled, open-label, phase II investigator-initiated trial (IIT) design.

**
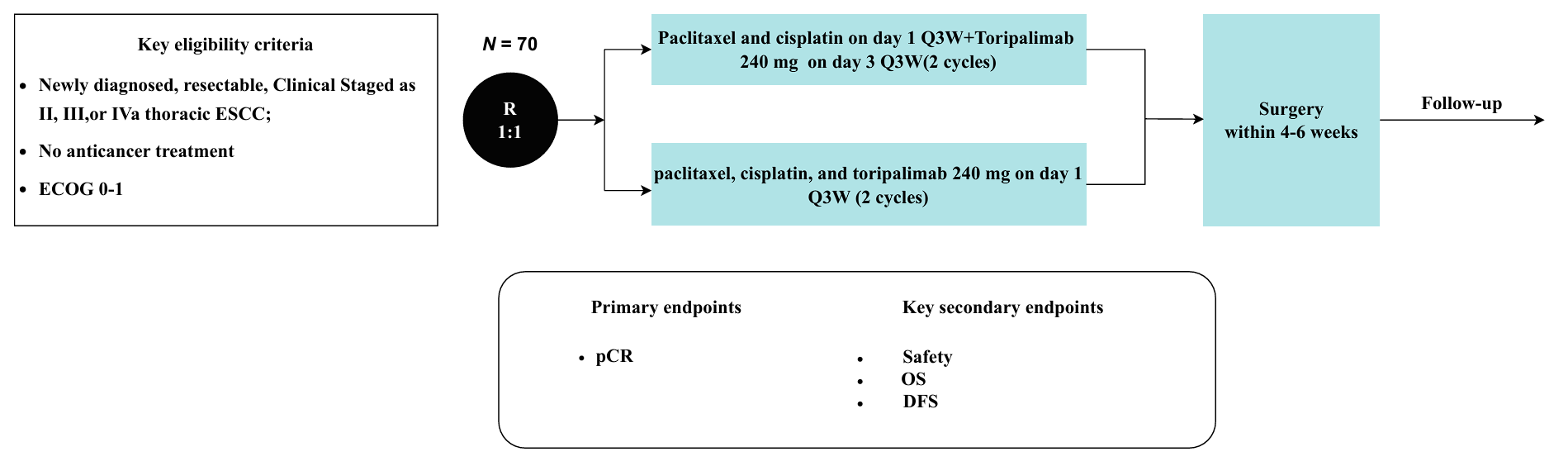
**

**eFigure 2.** Consort Flow Diagram

Graphical illustration of patient disposition during screening, neoadjuvant treatment, and curative resection.

**
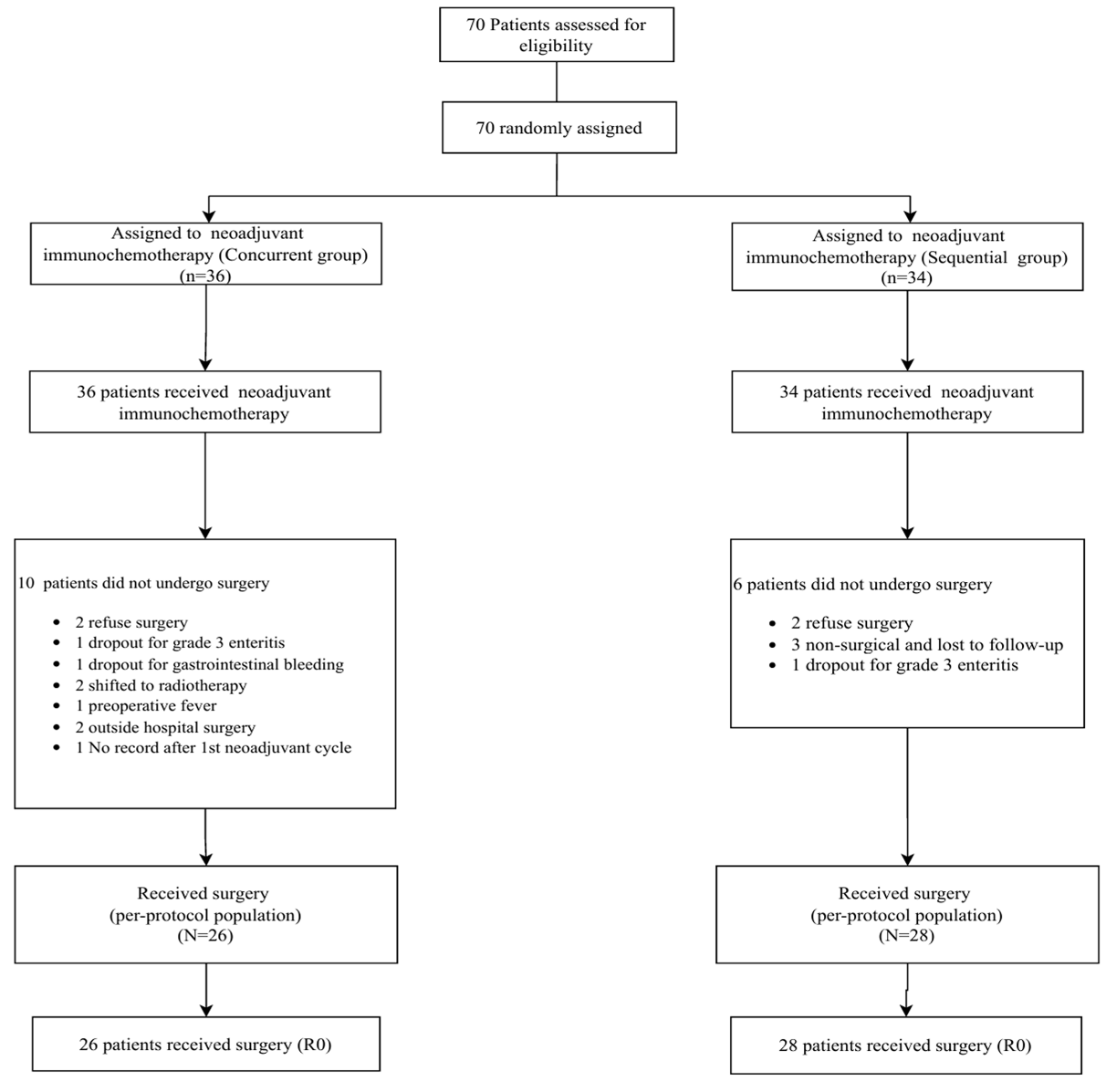
**

**eFigure 3. (A)** Overall survival (OS); (**B)** Disease-free survival (DFS) in the sequential vs concurrent groups


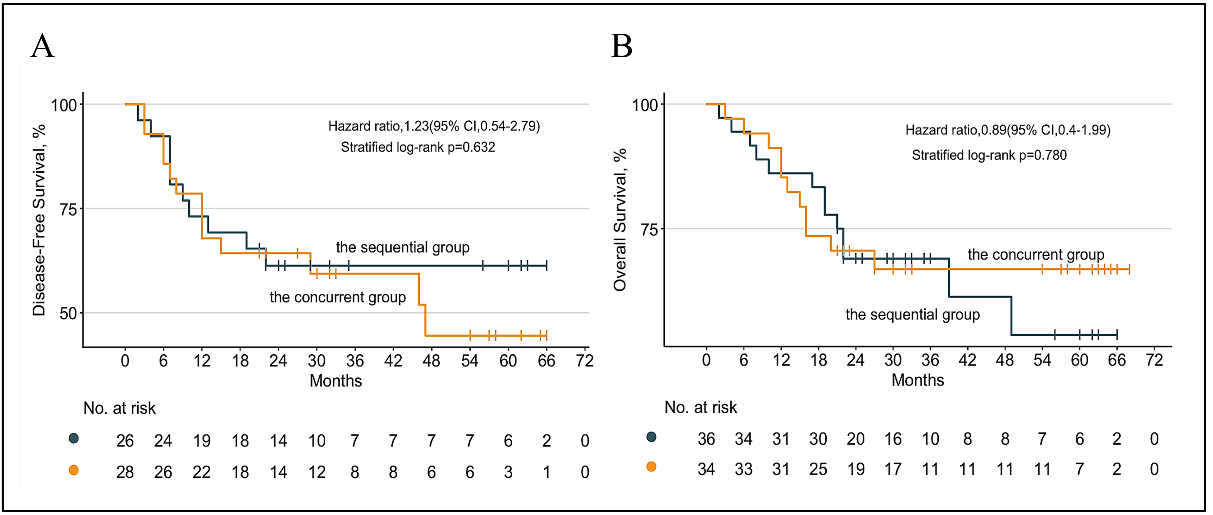


**eTable 1. Recurrence Pattern (N=54) and Reason for Death (N=70).**

| Variable | No. (%) |
| --- | --- |
| **Recurrence Pattern** | 54(100) |
| Recurrence | 12 (22.2) |
| LRR Only | 5(9.2) |
| DM Only | 6(11.1) |
| LRR And DM | 1 (1.8) |
| **The postoperative 30-day mortality rate** | 1 (1.8) |
| **Death Reason** | 70(100) |
| The concurrent Group  Myocarditis(Immune-Related) | 13(18.6)  1 (1.4) |
| Hyperglycemic Ketoacidosis  Postoperative Pneumonia | 1 (1.4)  1 (1.4) |
| Upper Gastrointestinal Hemorrhage | 1 (1.4) |
| Esophageal Fistula After Radiotherapy | 1 (1.4) |
| Cancer related death | 8(11.4) |
| The sequential Group | 11(15.7) |
| Gastric Hemorrhage | 1 (1.4) |
| Cancer related death | 8(11.4) |
|  |  |
| Abbreviations: LRR, locoregional recurrence; DM, distant metastasis. | |

**eTable 2.Compliance to Chemotherapy With Respect to Chemotherapy Cycles**

| Chemotherapy Cycles Received |  | No. (%) |
| --- | --- | --- |
| 0 |  | 0 |
| 1 |  | 5(7.1%) |
| 2 |  | 65(92.8%) |

**eTable 3.Overall Survival (OS) Rates of the The sequential Group and concurrent Groups at 1, 3, and 5 Years**

| Variable |  | The 1-year OS Rate  (95% CI) | The 3-year OS Rate  (95% CI) | The 5-year OS Rate  (95% CI) | *P* |
| --- | --- | --- | --- | --- | --- |
| S group | | 86.10(75.50 to 98.20) | 69.00(55.30 to 86.10) | 53.70(35.50 to 81.10) | 0.780 |
| C group | | 85.30 (74.20 to 98.10) | 66.90(52.50 to 85.10) | 66.9(52.50 to 85.10) |  |

**eTable 4.Disease Free Survival (DFS) Rates of the sequential and concurrent Groups at 1, 3, and 5 Years**

| Variable |  | The 1-year DFS Rate | The 3-year DFS Rate | The 5-year DFS Rate | *P* |
| --- | --- | --- | --- | --- | --- |
| S group | | 73.10(57.90 to 92.30) | 61.30(45.10 to 83.30) | 61.30(45.10 to 83.30) | 0.632 |
| C group | | 67.90 (52.60 to 87.60) | 59.30(43.20 to 81.50) | 44.50(26.70 to 74.20) |  |
